# Ecological and Health Risk Assessment of Potentially Toxic Elements in the Small Wetlands in the Baghrash Lake Basin, China

**DOI:** 10.3390/toxics14070547

**Published:** 2026-06-24

**Authors:** Mamattursun Eziz, Mireguli Ainiwaer

**Affiliations:** 1 College of Geographical Science and Tourism, Xinjiang Normal University, Urumqi 830054, China; mihray@xjnu.edu.cn; 2 Xinjiang Laboratory of Arid Zone Lake Environment and Resources, Xinjiang Normal University, Urumqi 830054, China

**Keywords:** small wetlands, PTEs, pollution, ecological risk, health risk

## Abstract

Despite their size, small wetlands play a vital role in maintaining ecosystem stability. To clarify the pollution levels as well as potential ecological and health risks of potentially toxic elements (PTEs) in small wetlands, 85 water samples were collected from small wetlands in the Baghrash Lake Basin (BLB) of China, and six PTEs (As, Cd, Cu, Hg, Pb, and Zn) were determined for their contents. The Nemerow integrated pollution index (NPI) was adopted to evaluate PTE pollution levels. The ecological risk index (RI) and USEPA health risk assessment model were further applied to quantify potential ecological and health risks of PTEs, respectively. The results revealed that PTEs in small wetlands showed a slight pollution level, with an average NPI value of 0.73. Meanwhile, the integrated ecological risk index of PTEs showed a low ecological risk level, with an average RI value of 23.041. Health risk assessment results demonstrated that the non-carcinogenic risk of PTEs in small wetlands remained at a negligible level, while the carcinogenic risk stayed within acceptable limits for both local population groups: children and adults. Among all detected PTEs, Hg was identified as the primary pollutant and major ecological risk factor, while As posed the highest relative potential health risk while overall risks remained acceptable. The findings of this study can provide a scientific basis for the environmental protection of small wetlands in the BLB.

## 1. Introduction

Wetlands, known as the “Kidney of the Earth”, refer to multifunctional transition zones bridging land and water ecosystems, which provide various ecological and economic benefits [1,2]. Wetlands perform several important ecosystem functions such as water supply [3], climate regulation [4], nutrient removal [5], flood regulation [6], carbon storage [7], landscape recreation [8], and provision of animal and plant habitats [9]. The water quality of wetland overlying water directly affects ecosystem health and human well-being [10]. Rapid population growth and extensive industrial and agricultural activities in recent decades have led to degradation of wetlands and contamination of wetland water with various potentially toxic elements (PTEs), significantly threatening human health and ecosystem stability [11]. In particular, environmental pollutants such as PTEs in wetlands have attracted increasing ecological attention because of their significant threat to human and environmental health [12].

Small wetlands are defined as natural or artificial wetland ecosystems that cover an area of <8 ha [13]. Natural small wetlands include ponds, small lakes, seasonal pools, streams, depressions, small rivers, springs, and ditches formed during long-term natural evolution. Artificial small wetlands comprise reservoirs, aquaculture ponds, paddy fields, and small wetlands in urban parks [14,15]. These small wetlands are critical for biodiversity conservation and global carbon cycle balance [12]. Small wetlands were previously not covered by conservation policies that govern larger water bodies. The “Resolution on Conservation and Management of Small Wetlands”, proposed by China, was adopted during the Ramsar Convention in 2018, and increasing attention has been given to small wetlands worldwide [12].

Potentially toxic elements are regarded as one of the most concerning pollutants in the surface environment [16]. The serious eco-environmental risk posed by PTEs is associated with their persistence, accumulation, toxicity, and non-biodegradability across ecological systems [17,18]. Even at low contents, PTEs may exert severe eco-environmental effects. Specifically, PTEs such as As, Cd, Hg, and Pb in the water environment pose serious ecological and health risks [19]. PTEs continuously migrate and transform among water, soil and atmospheric media, and eventually accumulate in various water bodies. Their accumulation disrupts the aquatic ecological balance and alters microbial community structures. Additionally, these pollutants enter the human body through food chains and impair the nervous and immune systems. Such contamination has become a global environmental problem with profound ecological and health implications [20,21,22]. Pollution of small wetlands by PTEs is a serious issue, as it threatens ecosystem stability and human health. Nevertheless, existing studies on PTE pollution in water bodies mainly focus on large lakes, groundwater and rivers. Research regarding PTE pollution and related risks in small wetlands remains rarely reported [23].

Arid zone small wetlands are an important component of arid ecosystems with distinct hydrological processes [12]. Despite their size, arid zone small wetlands exert an important effect in regulating the stability of desert ecosystems and supporting sustainable socio-economic development [12]. Arid zone small wetlands help alleviate water shortages by supplying irrigation water for both agriculture and natural ecosystems, while also sustaining plants and animals with a stable water supply and protecting biodiversity of desert ecosystems [24]. With the development of regional urbanization, industrialization, and agricultural intensification, arid zone small wetlands are currently facing significant challenges, such as reductions in area and number, deterioration of water quality, and ecological degradation [12]. However, it is imperative to protect small wetlands and ensure sustainable water use in arid zones. At present, the pollution risk of PTEs in arid zone small wetlands has not been extensively discussed, and the pollution and potential ecological/health risks of PTEs in arid zone small wetlands are unclear.

The BLB is a major pepper-producing area in the arid zone of Northwest China. This study aimed to (1) investigate the concentrations, pollution levels and ecological risks of six PTEs (As, Cd, Cu, Hg, Pb, Zn) in the overlying water of small wetlands in the BLB; and (2) evaluate the potential human health risks of these PTEs using the USEPA health risk assessment model. The research results are expected to provide a scientific reference for environmental protection and ecological security management of small wetlands in the BLB.

## 2. Materials and Methods

### 2.1. Study Area

**Figure 1:**
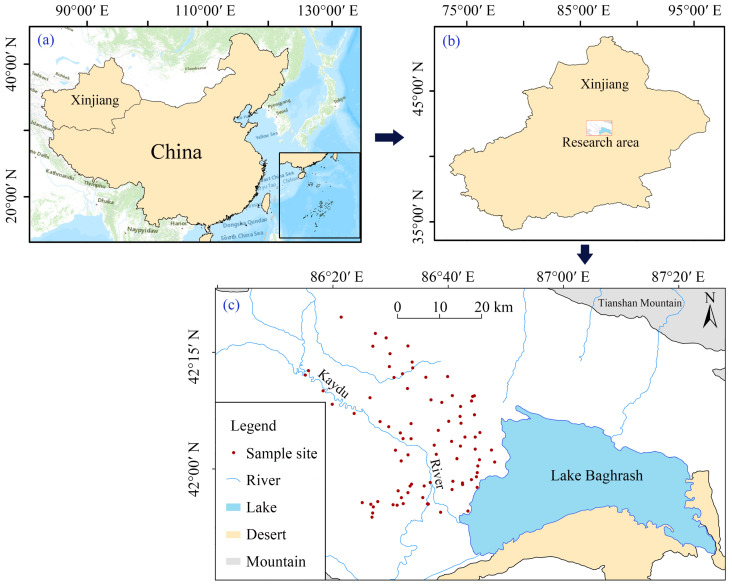
Locations of the BLB and wetland sampling sites. (**a**) Map of China; (**b**) Location of BLB; (**c**) Sample sites.

The BLB, located at 86°00′–87°00′ E and 41°50′–42°30′ N, lies in the northern Taklimakan Desert in the northwest arid belts of China (Figure 1). The BLB is the largest fishery production base and dominant pepper production region in Xinjiang, China [25]. Baghrash Lake, China’s largest inland freshwater lake, exerts an essential influence on the regional water cycle and provides important habitats and breeding grounds for migratory birds [25]. A warm temperate continental arid climate prevails in the BLB, with an altitude of 1050–1800 m, and annual average precipitation, evaporation, and temperature of 68.2 mm, 2200 mm, and 9.03 °C, respectively [25]. In recent years, under the impacts of oasis urbanization, agricultural intensification, industrial wastewater, and domestic sewage, the soil, surface water, and groundwater environments in the BLB have been affected by PTE pollution risks to varying degrees [26]. The regional geological setting is dominated by pre-Sinian ancient basement and Mesozoic–Cenozoic continental sedimentary sequences. The surrounding mountains are mainly composed of metamorphic rocks, granite and sedimentary bedrock, while the basin floor is covered by fluvial–lacustrine deposits, alluvium and diluvium transported primarily by the Kaidu River system [26].

### 2.2. Wetland Water Sampling

In May 2024, a total of 85 representative water samples were collected from the overlying water within small wetlands in the BLB, covering the main distribution areas of local small wetlands. A stratified random sampling strategy was adopted to ensure a uniform sample distribution along geographical, land-use and ecological gradients. The locations of small wetland sampling sites are also illustrated on the map in Figure 1. Before sampling, pre-prepared polyethylene plastic bottles were washed with deionized water and then rinsed three times with in situ wetland water. After washing, water samples were preserved in polyethylene bottles (500 mL). Ultrapure nitric acid (HNO_3_) was added on-site to adjust the pH of water samples to below 2 to stabilize PTEs. The 0.45 μm microporous membranes were then used to filter all small wetland water samples. After being sealed with sealing film, the samples were transported to the laboratory and stored at 4 °C.

### 2.3. Chemical Analysis

Six PTEs (As, Cd, Cu, Hg, Pb, and Zn) in small wetland overlying water were determined in this study. Among them, As and Hg levels in small wetlands were determined using an atomic fluorescence spectrophotometer (BAF–4000, Baode, Beijing, China) following the method described in HJ 694–2014 (Water quality–Determination of mercury, arsenic, selenium, bismuth and antimony–Atomic fluorescence spectrometry) [27]. The Cd, Cu, Pb, and Zn levels were measured using inductively coupled plasma mass spectrometry (ICP–MS, Perkin Elmer, Waltham, MA, USA), in accordance with HJ 700–2014 (Water quality–Determination of 65 elements–Inductively coupled plasma-mass spectrometry) [28]. The method detection limits (MDLs) of the above six PTEs were 0.30, 0.05, 0.08, 0.04, 0.09, and 0.67 μg/L, respectively. Notably, Cd in 75 water samples and Hg in 55 water samples were below the corresponding MDLs. Thus, only samples with detectable Cd and Hg were included in subsequent analyses.

### 2.4. Quality Control

Before analysis, wetland water samples were allowed to equilibrate to room temperature, and blank tests were conducted with distilled water to ensure the reliability of results. Quality control was carried out using blank samples and replicate samples for each wetland water sample. Quality control standards were applied to establish calibration curves for assessing the data obtained from wetland water samples. For each wetland water sample, reagent blanks and duplicate samples were examined in triplicate, and the average value was taken as the final content of PTEs. The recovery rates of all PTEs in wetland water samples were found to vary from 94.3% to 106.8%.

### 2.5. Nemerow Pollution Index (NPI)

The NPI method [29] was used to assess the PTE pollution level within overlying water in small wetlands in the BLB. It is calculated by the following equation:

(1) $$P_{i\;}={\;C}_{i}/S_{i}$$

(2) $$\mathrm{NPI}=\sqrt{\left({P_{\mathrm{imax}}}^{2}+{P_{\mathrm{iave}}}^{2}\right)/2}$$

in which *P_i_* refers to the single-factor pollution index of PTE *i* within overlying water in small wetlands; and *C_i_* and *S_i_* are the actual content and assessment standard of PTE *i* within small wetlands, respectively. In this research, Class III limit values specified in GB 3838–2002 (Environmental Quality Standards for Surface Water) [30] were used as the evaluation standard. *P_imax_* and *P_iave_* represent the maximum and mean *P_i_* levels, respectively. Table 1 presents the classification standards [29] for *P_i_* and NPI.

### 2.6. Ecological Risk Index (RI)

The RI developed by Håkanson [31] was utilized to evaluate the ecological risks of PTEs within small wetlands. RI can be determined below:

(3) $$E_{i}\;={\;P}_{i}\;\text{×}{\;T}_{i}$$

(4) $$\mathrm{RI}=\sum_{i=1}^{n}E_{i}$$

in which *E_i_* is the single ecological risk index for PTE *i* in small wetlands, while RI is the comprehensive ecological risk index of all PTEs; *P_i_* is the same as in Equation (1). *T_i_* indicates the toxic-response factor. For the above six PTEs, the toxic-response factors are 10, 30, 5, 40, 5, and 1, respectively [31]. The classification criteria [19,23] for ecological RI are given in Table 2.

### 2.7. Health Risk Assessment

#### 2.7.1. Exposure Analysis

The human health risk assessment model proposed by the U.S. Environmental Protection Agency (USEPA) [32] was adopted to estimate potential health risks associated with exposure to PTEs in small wetlands. Referring to the classification defined by the International Agency for Research on Cancer [33], target PTEs in small wetlands were divided into non-carcinogenic and carcinogenic types. Accordingly, the potential health risk assessment comprised two categories: non-carcinogenic and carcinogenic health risk [34]. Generally, PTEs within wetlands are mainly exposed through dermal contact and oral ingestion of water [12]. In the present study, the average daily doses (*ADD*) of PTEs in small wetlands were estimated via the two exposure pathways and used to assess non-carcinogenic and carcinogenic risks for local adults (≥21 years old) and children (0–21 years old) [32]. *ADD* is calculated as follows:

*ADD_ingest_* = (*C_i_* × IR × EF × ED)/(BW × AT) (5)

*ADD_dermal_* = (*C_i_* × SA × K_P_ × ET × EF × ED × 10^−3^)/(BW × AT) (6)

*ADD_total_* = *ADD_ingest_* + *ADD_dermal_* (7)

where *ADD_ingest_* and *ADD_dermal_* represent the average daily doses of overlying water within small wetlands via oral ingestion and dermal contact (μg/kg/day); and *C_i_* is the content of PTE *i* in small wetlands. Exposure parameters in the health risk assessment model are given in Table 3.

#### 2.7.2. Non-Carcinogenic Health Risk

The potential non-carcinogenic health risk induced by PTEs within small wetlands can be calculated as the hazard quotient (*HQ*) by Equation (8) [39]:

*HQ_i_* = *ADD*/*RfD_i_* (8)

where *RfD_i_* indicates the reference dose of PTE *i* in small wetlands. It describes an estimated daily exposure dose of a chemical that the general human population can ingest every day over an entire lifetime without suffering any observable adverse toxic health effects [32]. Considering the total non-carcinogenic health risks associated with the tested PTEs in small wetlands, the total non-carcinogenic risk index (HI) can be calculated as follows:

(9) $$\mathrm{HI}\;=\;{\mathrm{HQ}}_{\mathrm{ingest}}\;+\;{\mathrm{HQ}}_{\mathrm{dermal}}$$

The threshold value of HI is 1.0; *HQ* or HI ≤ 1 indicates no significant non-carcinogenic risk from PTEs in small wetlands, while *HQ* or HI > 1 indicates a potential non-carcinogenic risk concern [40,41].

#### 2.7.3. Carcinogenic Health Risk

The potential carcinogenic health risk induced by PTEs within small wetlands can be calculated as the carcinogenic risk index (*CR*) by Equation (10):

*CR_i_* = *ADD* × *SF_i_* (10)

where *SF_i_* is the carcinogenic slope factor of PTE *i* in small wetlands, representing the excess lifetime cancer probability caused by long-term daily exposure to carcinogens [32]. Considering the total carcinogenic health risks associated with tested PTEs within small wetlands, the total carcinogenic risk index (TCR) of all PTEs is calculated using the following formula [42]:

(11) $$\mathrm{TCR}\;=\;{\mathrm{CR}}_{\mathrm{ingest}}\;+\;{\mathrm{CR}}_{\mathrm{dermal}}$$

Carcinogenic health risk effects from PTEs in small wetlands are considered negligible when CR or TCR < 1 × 10^–6^, or tolerable within 1 × 10^–6^~1 × 10^–4^, and TCR > 1 × 10^–4^ indicates a potential carcinogenic health risk effect requiring urgent attention [39,41]. Table 4 displays *RfD* and *SF* values of analyzed PTEs under different exposure pathways [12,43].

## 3. Results and Discussion

### 3.1. PTE Levels in Small Wetlands

Table 5 presents the summary statistics of tested PTEs within overlying water in small wetlands in the BLB and the Class III screening values from the Environmental Quality Standards for Surface Water (GB 3838–2002). The contents of As, Cd, Cu, Hg, Pb, and Zn within small wetlands ranged from 0.30 to 13.30, 0.06 to 0.80, 0.08 to 57.30, 0.06 to 0.57, 0.09 to 12.70, and 0.67 to 77.50 μg/L, with average values of 2.38, 0.16, 15.58, 0.16, 2.23, and 37.52 μg/L, respectively. Except for Hg, the average contents of the other five PTEs were lower than the Class III screening value in GB 3838–2002. Notably, the average content of Hg was 1.6 times the standard limit, with an exceedance ratio of 73.33%, and the maximum content of Hg reached 5.7 times the standard value. Based on these results, wetlands within the BLB were affected by PTEs to varying degrees, while Hg was particularly more abundant in these wetlands. The pH of water samples ranged from 6.84 to 8.36 (average 7.43), indicating neutral to alkaline conditions. Electrical conductivity ranged from 423 to 5820 (average 1953.94) μS/cm.

Standard deviations (St.D) for contents of PTEs in small wetlands were relatively high (Table 5), indicating that PTE contents varied greatly among sampling sites. The coefficient of variation (CV) reflects the spatial heterogeneity of PTE contents in small wetlands. A CV > 0.5 indicates strong variability and potential point-source pollution [12]. Based on this criterion and the calculated CV values of wetland water samples, except for Zn, all other five PTEs had CV > 0.5, indicating strong spatial heterogeneity and possible anthropogenic inputs. Spatially, Zn exhibited moderate variability. The elevated CV values of these PTEs in small wetlands suggest the anthropogenic influence [19].

### 3.2. Pollution of PTEs in Small Wetlands

**Figure 2:**
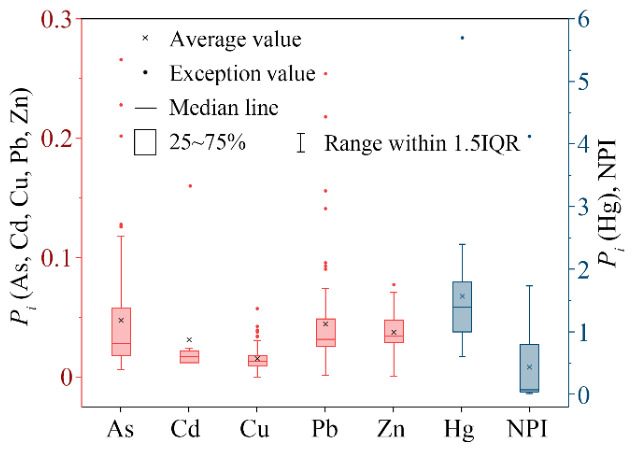
Box plot of pollution levels of PTEs in small wetlands in the research area.

The PTE pollution levels within overlying water in small wetlands of the BLB were classified according to *P_i_* and NPI values (Figure 2). Based on the average *P_i_* level, as shown in Figure 2, the pollution grade of *P_i_* for all PTEs in small wetlands decreases as follows: Hg (1.567) > As (0.048) > Pb (0.045) > Zn (0.038) > Cd (0.031) > Cu (0.016), where larger *P_i_* values indicate higher pollution levels. According to the *P_i_* classification standard [29] and the average *P_i_* values for PTEs within small wetlands, the studied wetlands are lightly polluted by Hg and not polluted by As, Cd, Cu, Pb, or Zn. More specifically, Hg in 6.67%, 20.0%, 63.33%, 6.67%, and 3.33% of water samples falls into the no-pollution, mild, low, moderate, and heavy pollution categories, respectively.

The NPI values of PTEs in small wetlands range from 0.01 to 4.121 (average 0.73), corresponding to a mild pollution level according to the NPI standard. Among the 85 small wetland samples, 69.41% exhibit a no-pollution level, whereas 11.76%, 17.65%, and 1.18% exhibit mild, low, and heavy pollution levels, respectively.

### 3.3. Ecological Risk Induced by PTEs Within Small Wetlands

**Figure 3:**
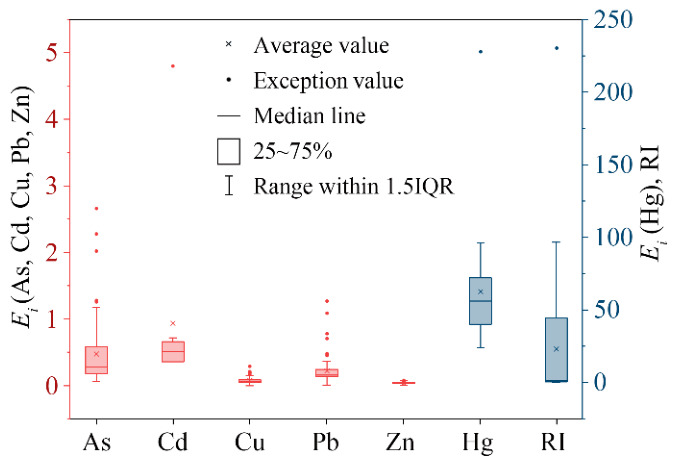
Box plot of ecological risk levels of PTEs in small wetlands inside the research area.

As shown in Figure 3, the ecological risk grade of *E_i_* for all PTEs in small wetlands decreases as follows: Hg (62.667) > Cd (0.936) > As (0.475) > Pb (0.223) > Cu (0.078) > Zn (0.038), where larger *E_i_* values indicate higher ecological risk levels. According to the *E_i_* classification standard [31] and the average *E_i_* values for PTEs in small wetlands, except for Hg, the other five PTEs resulted in a low ecological risk. For Hg, small wetlands within the research area posed a moderate ecological risk on average and a high ecological risk at the maximum (*E_i_* = 228.00). More specifically, the proportions of low, moderate, considerable, and high ecological risk sites for Hg in small wetlands were 13.33%, 70.01%, 13.33%, and 3.33%, respectively.

RI values for PTEs within small wetlands range from 0.235 to 230.371 (average 23.041), corresponding to a low ecological risk as defined by the RI classification standard. Among the 85 small wetland water samples, 98.82% show a low ecological risk level, while 1.18% show a moderate ecological risk level. Except for Zn, all Ei and RI values had CV > 0.5, indicating strong spatial variability and revealing significant spatial differences in ecological risks of PTEs in small wetlands within the research area.

Based on these results, Hg was the main pollution factor and primary ecological risk factor in small wetlands within the BLB. Notably, Hg is a highly toxic element known for its neurotoxicity, genotoxicity, bioaccumulation, and persistence, and is classified as a Group 3 carcinogen by IARC [44]. Thus, pollution and ecological risks induced by Hg within small wetlands in the research area require further attention.

### 3.4. Possible Non-Carcinogenic Health Risk Related to PTEs Within Small Wetlands

Table 6 summarizes the calculated hazard quotient index (*HQ*) of PTEs within overlying water in small wetlands for two population groups, adults and children, based on two exposure pathways: ingestion and dermal contact. Under the ingestion pathway, the non-carcinogenic hazard quotients (*HQ_ingest_*) for individual PTEs in small wetlands ranged from 3.61 × 10^–3^ (Zn, adults) to 2.62 × 10^–1^ (As, children). *HQ_ingest_* values of PTEs in small wetlands for children and adults followed a similar trend. Specifically, the average HQingest values of PTEs in small wetlands showed a decreasing order: As > Pb > Hg > Cu > Cd > Zn in both adult and child groups.

In contrast, the non-carcinogenic risk caused by PTEs within small wetlands through dermal contact (*HQ_dermal_*) was several orders of magnitude lower. Specifically, the average *HQ_dermal_* values of PTEs in small wetlands exhibited a decreasing order: Cd > Hg > Cu > As > Pb > Zn in adults, whereas they were ordered as Cd > As > Hg > Cu > Pb > Zn in children. The *HQ_dermal_* for individual PTEs ranged from 5.79 × 10^–5^ (Zn, adults) to 1.02 × 10^–2^ (As, children). The above analysis indicates that oral ingestion serves as the main pathway for potential non-carcinogenic health risk caused by the tested PTEs within small wetlands in the BLB. Overall, the average *HQ_total_* values of PTEs in small wetlands ranged from 3.67 × 10^–3^ (Zn, adults) to 2.68 × 10^–1^ (As, children), and followed a decreasing order: As > Pb > Cd > Hg > Cu > Zn in adults, while they were ordered as As > Pb > Hg > Cd > Cu > Zn in children.

**Figure 4:**
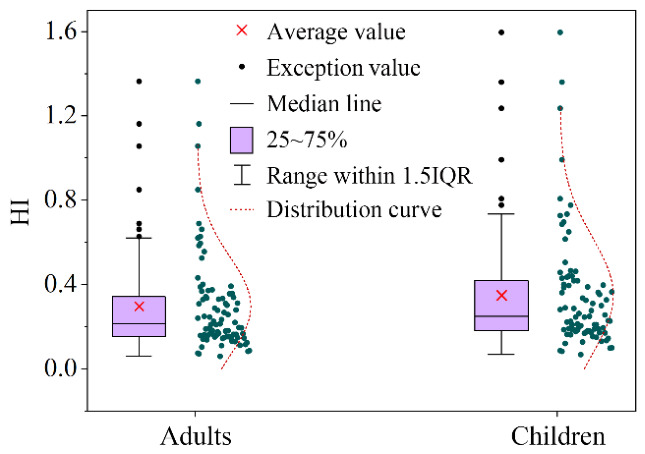
Box plot of the total non-carcinogenic risk index (HI) of PTEs in small wetlands.

As illustrated in Figure 4, the total HI of PTEs within small wetlands ranged from 0.059 to 1.363 (average 0.29) for adults, and 0.086 to 1.597 (average 0.34) for children. These results indicate that children exhibit a higher non-carcinogenic health risk from ingestion-related exposure to PTEs in small wetlands. This finding is consistent with conclusions from recent studies [34,45,46], which also report that PTEs in water bodies have greater non-carcinogenic effects on children’s health than on adults. This is primarily attributed to the lower average body weight (BW) and non-carcinogenic average exposure time (AT) of children, leading to higher estimated daily doses (*ADD*).

However, the average HI values of PTEs in small wetlands for both adult and child groups are < 1, indicating a negligible overall non-carcinogenic risk according to the HQ and HI classification standards [40,41]. It should be noted that PTEs in 3.53% of small wetland samples posed unacceptable non-carcinogenic health risk levels (HI > 1) in both adult and child groups. This suggests that PTEs in some small wetlands within the BLB may present localized non-carcinogenic health risks.

Overall, in adults, total *HQ* values for As, Cd, Cu, Hg, Pb, and Zn within small wetlands accounted for 69.57%, 0.42%, 5.54%, 2.62%, 20.02%, and 1.82% of the total non-carcinogenic risk (HI), respectively. In children, these values accounted for 69.70%, 0.64%, 5.51%, 2.71%, 19.63%, and 1.81% of HI, respectively. This indicates that As is the primary contributor to the potential non-carcinogenic health risk induced by PTEs within small wetlands. However, a relatively higher non-carcinogenic health risk was observed in children compared with adults, reflecting the combined influence of differences in body weight and exposure time between the two population groups.

### 3.5. Possible Carcinogenic Health Risk Caused by PTEs Within Small Wetlands

Arsenic and Cd are classified as carcinogenic PTEs according to IARC [47]. Therefore, the carcinogenic health risks of these two PTEs in small wetlands in the BLB were evaluated. Table 7 summarizes the calculated carcinogenic risk index (*CR*) of As and Cd within small wetlands for adult and child groups according to two exposure pathways: ingestion and dermal contact. As shown in Table 7, under the ingestion pathway, the carcinogenic risk index (*CR_ingest_*) for individual PTEs in small wetlands ranged from 2.69 × 10^–6^ (Cd, children) to 4.41 × 10^–5^ (As, adults). The average *CR_ingest_* value of As was higher than that of Cd. Meanwhile, the *CR_dermal_* for individual PTEs ranged from 1.67 × 10^–9^ (Cd, children) to 5.75 × 10^–7^ (As, adults). It can be concluded that oral ingestion represents the main pathway for the potential carcinogenic health risk induced by the tested PTEs within small wetlands in the BLB. Similarly, the average *CR_dermal_* values of As were higher than those of Cd in small wetlands. Overall, the average *CR_total_* values of PTEs in small wetlands ranged from 2.69 × 10^–6^ (Cd, children) to 4.46 × 10^–5^ (As, adults), and followed a decreasing order: As > Cd for both adults and children.

**Figure 5:**
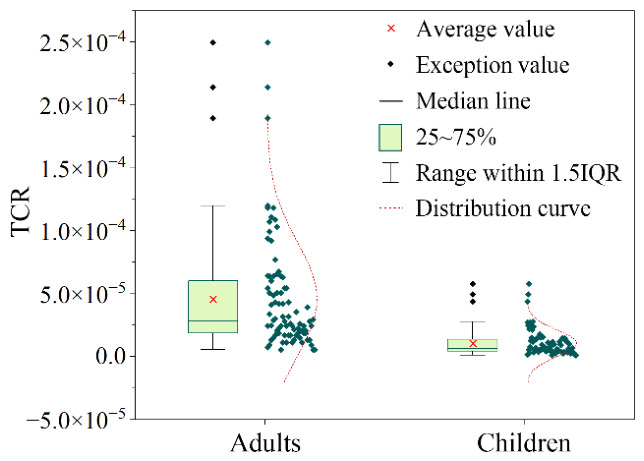
Box plot of the total carcinogenic risk index (TCR) of PTEs in small wetlands.

From Figure 5, the TCR of the two PTEs in small wetlands ranged from 5.64 × 10^–6^ to 2.50 × 10^–4^ for adults, and 1.30 × 10^–6^ to 5.78 × 10^–5^ for children, with average values of 4.60 × 10^–5^ and 1.07 × 10^–5^ for the adult and child groups, respectively.

These results indicate that children exhibit a higher carcinogenic health risk from ingestion-related exposure to PTEs in small wetlands. However, this observation is inconsistent with some previous studies [12,34], which report that PTEs in water bodies may pose higher carcinogenic risks to adults than to children. This difference is primarily attributed to a larger body weight (BW), higher daily water intake (IR), longer exposure duration (ED), and larger skin surface area (SA) in adults, which influence the estimated daily dose (*ADD*).

However, the average TCR values for the two PTEs within small wetlands in adult and child groups fall within 1 × 10^–6^~1 × 10^–4^, indicating acceptable carcinogenic risk levels according to the TCR classification standard [39,41] for both population groups. It should be noted that PTEs in 11.76% of small wetland samples may pose unacceptable levels (TCR > 1 × 10^–4^) of potential carcinogenic health risk for adults. Therefore, PTEs in some small wetlands within the research area may present carcinogenic health risks for adults.

Overall, the total *CR* values for As and Cd in small wetlands accounted for 97.32% and 2.68% of the TCR, respectively, for adults. For children, the total *CR* values of these two PTEs accounted for 97.34% and 2.66% of TCR, respectively. This indicates that As is the primary contributor to the potential carcinogenic health risk induced by PTEs within small wetlands in the BLB. A relatively higher carcinogenic health risk caused by PTEs was observed in adults compared with children, reflecting the combined influence of differences in body weight, daily water consumption, exposure duration, and exposed skin surface area between the two age groups [34].

According to the findings of the present study, arsenic (As) can be identified as the primary contributor to both carcinogenic and non-carcinogenic health risks within small wetlands in the BLB. Arsenic in the surface environment is harmful to human and animal health even at low concentrations due to its high toxicity [19,48]. Therefore, water with elevated As represents a critical environmental problem globally, as As is highly toxic and can accumulate in the body [49]. It is worth noting that arsenic and its compounds are major pollutants in groundwater and surface water in China and are listed in the “List of Toxic and Hazardous Water Pollutants (First Batch)” [19]. As is also categorized by IARC [47] as a Class I carcinogen. Arsenic can migrate and transform across different ecosystems, threatening overall ecosystem health [50]. The results of this study highlight the non-negligible health risks associated with As exposure to humans in small wetlands within the BLB, considering its strong hazardous effects. Therefore, effective control of As pollution in small wetlands is necessary to minimize health risks while ensuring ecosystem safety. The findings of this study provide further insights into water management strategies for small wetlands within the BLB and in other arid zones. Future research can focus on pollutant source apportionment, spatial distribution characteristics and comparative studies in other arid areas.

## 4. Conclusions

In this research, the contents of As, Cd, Cu, Hg, Pb, and Zn in small wetlands in the BLB were analyzed. In addition, pollution levels and the ecological and health risks induced by these PTEs in small wetlands were evaluated. According to the results, the average Hg content in small wetlands exceeded the evaluation criteria (GB 3838–2002) by 1.60 times. Moreover, small wetlands within the research area were lightly polluted by Hg, and the NPI values of PTEs ranged from 0.015 to 4.121 (average 0.73), corresponding to a slight pollution level. Furthermore, the ecological risk level of PTEs in small wetlands decreased in the order Hg > Cd > As > Pb > Cu > Zn, with Hg posing a moderate ecological risk. Therefore, Hg can be regarded as the major pollution and ecological risk factor within small wetlands in the BLB. Based on the health risk evaluation results, the six PTEs in small wetlands posed negligible non-carcinogenic health risks and acceptable carcinogenic health risks to both children and adults. In addition, oral ingestion represents the main pathway for the potential health risk induced by PTEs in small wetlands. Overall, arsenic poses the most significant carcinogenic and non-carcinogenic health risk among the investigated PTEs in small wetlands in the BLB. Regular monitoring of PTEs in small wetlands is recommended. The results of this study provide a scientific basis for environmental protection and ecological security of arid zone small wetlands.

## Figures and Tables

**Table 1:** Classification standard of the Nemerow Pollution Index.

Pollution Grade	*P_i_*	NPI
No pollution	*P_i_* ≤ 0.7	NPI ≤ 0.7
Slight pollution	0.7 < *P_i_* ≤ 1	0.7 < NPI ≤ 1
Low pollution	1 < *P_i_* ≤ 2	1 < NPI ≤ 2
Moderate pollution	2 < *P_i_* ≤ 3	2 < NPI ≤ 3
Heavy pollution	*P_i_* > 3	NPI > 3

**Table 2:** Classification standard of the *E_i_* and RI.

Risk Degree	*E_i_*	RI
Low risk	*E_i_* ≤ 40	RI ≤ 150
Moderate risk	40 < *E_i_* ≤ 80	150 < RI ≤ 300
Considerable risk	80 < *E_i_* ≤ 160	300 < RI ≤ 600
High risk	160 < *E_i_* ≤ 320	600 < RI ≤ 1200
Extremely high risk	*E_i_* > 320	RI > 1200

**Table 3:** Exposure parameters of the health risk assessment.

Physical Meaning (Parameter)	Unit	Reference Value		Source
		Adults	Children	
Ingestion rate (IR)	L/day	1.8	0.7	[35]
Exposure frequency (EF)	day/year	365	365	[32]
Exposure duration (ED)	years	30	6	[32]
Body weight (BW)	kg	62.4	21.2	[36,37]
Average time (AT)	days	10,950 (non-carcinogenic)	2190 (non-carcinogenic)	[32]
		25,500 (carcinogenic)	25,500 (carcinogenic)	
Skin surface area (SA)	cm^2^	16,600	12,000	[38]
Skin permeability coefficient (K_P_)	cm/h	As, Cd, Cu, Hg: 0.001; Pb: 0.0001; Zn: 0.0006		[12]
Exposure time (ET)	h/day	0.58	0.58	[38]

**Table 4:** *RfD* and *SF* values for PTEs.

PTEs	*RfD* (μg/kg/d)		*SF* (μg/kg/d)^−1^	
	Ingest	Dermal	Ingest	Dermal
As	0.3	0.1	1.5 × 10 ^−3^	3.66 × 10 ^−3^
Cd	0.5	0.5	6.1 × 10 ^−3^	3.8 × 10 ^−4^
Cu	40	12	/	/
Hg	0.3	0.021	/	/
Pb	1.4	1.4	/	/
Zn	300	10	/	/

Notes: “/” indicates no relevant data.

**Table 5:** Descriptive statistics of contents of PTEs in small wetlands.

PTEs	As	Cd	Cu	Hg	Pb	Zn
Minimum (μg/L)	0.30	0.06	0.08	0.06	0.09	0.67
Maximum (μg/L)	13.30	0.80	57.30	0.57	12.70	77.50
Average (μg/L)	2.38	0.16	15.58	0.16	2.23	37.52
Median (μg/L)	1.40	0.09	13.20	0.14	1.58	34.30
Standard deviation (μg/L)	2.39	0.23	9.85	0.09	1.95	14.49
Coefficient of variation (CV)	1.00	1.44	0.63	0.56	0.88	0.37
* National standard (μg/L)	50	5	1000	0.1	50	1000
Exceedance ratios (%)	0	0	0	73.33	0	0

Notes: * Environmental Quality Standards for Surface Water (GB 3838–2002) (Class III).

**Table 6:** The hazard quotient of PTEs in small wetlands in the BLB.

PTEs	*HQ_ingest_*		*HQ_dermal_*		*HQ_total_*	
	Adults	Children	Adults	Children	Adults	Children
As	2.29 × 10^–1^	2.62 × 10^–1^	1.26 × 10^–4^	6.34 × 10^–3^	2.29 × 10^–1^	2.68 × 10^–1^
Cd	9.00 × 10^–3^	1.03 × 10^–2^	1.73 × 10^–3^	1.02 × 10^–2^	1.07 × 10^–2^	2.05 × 10^–2^
Cu	1.12 × 10^–2^	1.29 × 10^–2^	2.00 × 10^–4^	4.26 × 10^–4^	1.14 × 10^–2^	1.33 × 10^–2^
Hg	1.51 × 10^–2^	1.72 × 10^–2^	1.01 × 10^–3^	2.14 × 10^–3^	1.61 × 10^–2^	1.94 × 10^–2^
Pb	4.59 × 10^–2^	5.25 × 10^–2^	8.18 × 10^–5^	1.74 × 10^–4^	4.60 × 10^–2^	5.27 × 10^–2^
Zn	3.61 × 10^–3^	4.13 × 10^–3^	5.79 × 10^–5^	1.23 × 10^–4^	3.67 × 10^–3^	4.25 × 10^–3^

**Table 7:** The carcinogenic risk index of PTEs in small wetlands in the BLB.

PTEs	*CR_ingest_*		*CR_dermal_*		*CR_total_*	
	Adults	Children	Adults	Children	Adults	Children
As	4.41 × 10^–5^	1.01 × 10^–5^	5.75 × 10^–7^	2.45 × 10^–7^	4.46 × 10^–5^	1.03 × 10^–5^
Cd	1.18 × 10^–5^	2.69 × 10^–6^	3.92 × 10^–9^	1.67 × 10^–9^	1.18 × 10^–5^	2.69 × 10^–6^

## Data Availability

The data presented in this study are available on request from the corresponding author.

## Abbreviations

The following abbreviations are used in this manuscript:

PTEs	Potentially toxic elements
BLB	Baghrash Lake Basin
NPI	Nemerow Pollution Index
RI	Ecological risk index
*ADD*	Average daily doses
*HQ*	Hazard quotient
HI	Non-carcinogenic risk index
*CR*	Carcinogenic risk index
TCR	Total carcinogenic risk index
*SF*	Carcinogenic slope factor
*RfD*	Reference dose
IR	Ingestion rate
EF	Exposure frequency
ED	Exposure duration
BW	Body weight
AT	Average time
SA	Skin surface area
K_P_	Skin permeability coefficient
ET	Exposure time
